# Central corneal thickness changes following manual small incision cataract surgery versus phacoemulsification for white cataract

**Published:** 2019

**Authors:** Pipat Kongsap

**Affiliations:** * Department of Ophthalmology, Prapokklao Hospital, Thailand

**Keywords:** manual small incision cataract surgery, white cataract, phacoemulsification, central corneal thickness, endothelial cell loss

## Abstract

**Aim:** To assess the central corneal thickness (CCT) and endothelial cell loss after manual small-incision cataract surgery and phacoemulsification in patients with white cataract.

**Material and methods:** This is a comparative, prospective, non-randomized study on 42 patients with white cataract, who underwent cataract surgery. The patients were divided into manual small-incision cataract surgery (21 eyes, MSICS group) and phacoemulsification cataract surgery group (21 eyes, phaco group). The endothelial cell density (ECD), central cornea thickness (CCT), and corrected distance visual acuity (CDVA) were evaluated at 1 day, 1 week, 4 weeks, and 3 months postoperatively. The results of 20 cases of nuclear sclerosis grade II-III (LOCS III) who underwent phacoemulsification by the same surgeon were also compared. Propensity scoring was used to adjust for confounding by selection bias.

**Results:** The CCT increased after surgery in both groups. The thickness was greater in the phaco group on first day postoperatively (73 µ increase in MSICS group and 138 µ in phaco group, p=0.008) and it returned to preoperative levels 1 month postoperatively. The endothelial cell loss was lower in the MSICS group at 3 months postoperatively (11.8% in MSICS group and 15.8% in phaco group, p=0.111). The CDVA was not different in both groups at 1 week and 4 weeks postoperatively (p>0.05).

**Conclusions:** Manual small-incision cataract surgery for white cataract provided less central corneal thickness changes compared to conventional phacoemulsification.

**Abbreviations:** CCT = central corneal thickness; ECD = endothelial cell density; CDVA = corrected distance visual acuity; APT = absolute phacoemulsification time; EPT = effective phacoemulsification time; MSICS = Manual small-incision cataract surgery in white cataract; Phaco II = Phacoemulsification in white cataract; Phaco I = phacoemulsification in NS 2 + Cataract; Phaco = Phacoemulsification in white cataract; APACRS = Asia-Pacific Association of Cataract and Refractive Surgeons

## Introduction

Cataract is one of the most common causes of blindness on all continents [**1**-**7**]. Various surgical treatments are available to help patients recover from the disease. Phacoemulsification provides better visual outcomes and risks fewer complications than ECCE [**8**-**11**]. However, surgery is difficult on a cataract that has become hypermature and cloudy (a white cataract) and is likely to cause postoperative complications, such as posterior capsule rupture [**12**-**13**] or corneal edema [**13**,**14**]. A comparative study of manual small-incision cataract surgery (MSICS) and phacoemulsification in 108 cataract patients found a 9-micron increase in corneal thickness in patients receiving MSICS and a 70-micron increase in corneal thickness in patients receiving phacoemulsification [**15**]. However, there are no published studies comparing these two methods for white cataract surgery.

The objective of this study was to compare central corneal thickness and endothelial cell loss resulting from hypermature cataract surgery using MSICS versus phacoemulsification.

## Material and Methods

The research design used in this study was a non-randomized clinical trial. The study was approved by the ethics committees of Prapokklao Hospital.

**Population: ** Cataract patients who underwent surgery in the ophthalmology division of Prapokklao Hospital between May 2016 and March 2017.

**Fig. 1:**
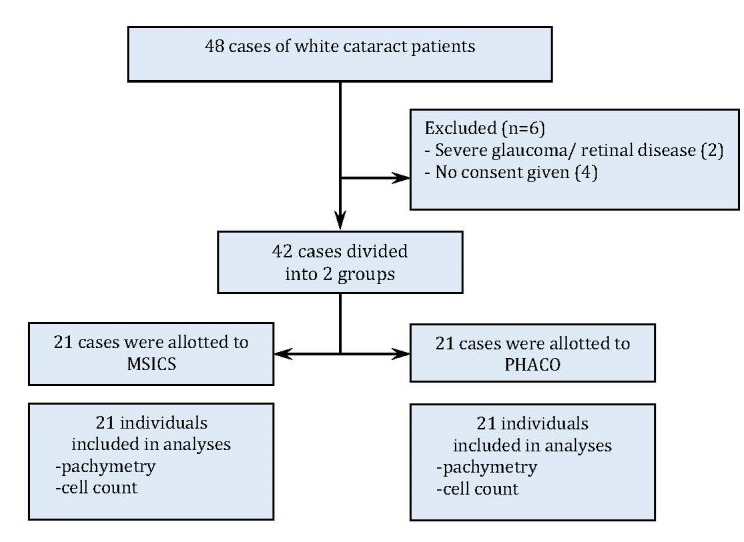
Flow diagram of study design

**Participants:** White cataract patients who received treatment by means of manual small incision cataract surgery or phacoemulsification (**Fig. 1**), selected based on the following inclusion and exclusion criteria:

**Inclusion criteria**

- White cataract patients with no zonular dialysis.

**Exclusion criteria**

- Cataract patients with concurrent diseases, such as glaucoma, retinal detachment, or diabetic retinopathy.

- Cataract patients with a cataract attributed to an accident.

**Sample size calculation**

A comparison by Jain [**15**] of MSICS versus phacoemulsification in patients with moderate cataract hardness (nuclear sclerosis 2+) showed an average corneal thickness of 541 ± 39 microns in patients who had undergone MSICS and 581 ± 60 microns in patients who had undergone phacoemulsification at a significant level of 0.05 (two-ways) and a statistical power of 80%. The samples were derived from 20 cataract patients in each group. The present research similarly examined standards of cataract surgery (nuclear sclerosis 2+) performed by the same surgeon on 20 patients, using these patients as a control group to evaluate surgical performance.

**Sampling methods**

Voluntary patients participating in the program received an explanation from a physician and signed a consent form to participate in the program. The patients underwent cataract surgery by means of manual small incision cataract surgery or phacoemulsification alternately without randomization (**Fig. 1**). The doctors collected patient and surgery details, such as age, gender, cataract hardness, and preoperative eyesight level, which were permitted to vary among patients in either group. A propensity score was used to estimate the likelihood or the probability of being assigned to each treatment arm.

**Surgical methods**

- Patients in both groups received an anesthetic (retrobulbar anesthesia). MSICS was performed using the modified Blumenthal technique. A scleral tunnel incision was made, capsulorhexis was performed, and viscoelastic material was injected into the anterior chamber. The lens was extracted and an intraocular lens was implanted in the capsular bag.

- Phacoemulsification was performed using Stellaris (Bausch & Lomb). A clear corneal incision was made and capsulorhexis was performed. The lens was emulsified and then replaced with an intraocular lens.

**Postoperative care**

Both groups of patients received the same postoperative care according to existing standards. After surgery, they received Dexoph eye drops and returned for follow-up checks after 1 day, 7 days, 1 month, and 3 months.

**Observation and assessment**

The following patient data were collected: age, gender, visual acuity, corneal thickness, endothelial cell count, and intraoperative and postoperative complications. Data were collected using the following parameters and techniques:

- Data on phacoemulsification were collected using the parameters of ultrasound power, absolute phacoemulsification time (APT), and effective phacoemulsification time (EPT);

- Surgery time measured the amount of time from the beginning of the surgery until the removal of the speculum;

- Corneal thickness was measured using a pachymeter (micron) on the 1st, 7th day and 1 month after surgery;

- Endothelial cell loss was measured using a specular microscope 1 month and 3 months prior to the day of surgery;

- Complications included posterior capsule rupture, vitreous loss, hyphema, iritis, lens drop, increased IOP, corneal edema, etc.

**Data Analysis**

Data analysis was conducted using a statistical program. A p-value less than 0.05 indicated statistical significance. Corneal thickness and endothelial cell loss were compared using a t-test and visual acuity and complications were compared using the Fisher Exact test.

## Results

The study included 42 participants, 21 of whom underwent MSICS and 21 phaco. The average age of the patients was 68 years (ranging from 40–83 years). Fifteen patients were males (35.7%). Patients’ general data, such as age, gender, and preoperative visual acuity were not significantly different (**Table 1**). 

**Table 1:** Patient baseline characteristics

Patient characteristics	MSICS (n= 21)		MSICS (n= 21)		
	n	%	n	%	p-value
Gender					
Male	7	44.6	8	53.3	
Female	14	48.2	13	51.8	0.747
Age (year)					
Mean ± sd	62.3 ±4.0		68.6 ±1.9		0.083
Pre-op VA <6/ 60	21	100	21	100	
Laterality (R)	11	47.8	12	52.2	1.000
Duration of Sx (min) Mean+-SD	13.2 ±0.6		13.2 ±0.6		0.086
Nuclear opacity					
NS ≤ 3+	16	72.6	13	61.9	0.202
NS 4+	5	23.8	8	38.1	

The data in **Table 2** represent the surgeon’s standard performance when conducting phacoemulsification on cataract patients with moderately hardened lenses. The surgeon spent 9.9 minutes performing the phaco surgery. The ultrasound power was 24.4%. The absolute phacoemulsification time (APT) was 50.4 seconds and the effective phacoemulsification time (EPT) was 12.2 seconds. By 1 day after the surgery, the cornea thickened by an average of 67 microns, and after 3 months, endothelial cell loss averaged 8.4%.

**Table 2:** Comparative clinical outcomes of phacoemulsification in normal cataract and white cataract by the same surgeon

	Phaco I (N=20)	Phaco (N=21)	p-value
Mean age	68.4 ± 2.24	68.6 ± 1.93	0.94
Female sex, n (%)	7 (35.0)	13 (61.9)	0.085
Operation time (Min)	9.9 ± 0.36	16.5 ± 12.3	0.009
Mean US power	24.5 ± 0.94	30.1 ± 1.5	0.004
Mean APT(s) ± SD	50.4 ± 3.35	95.9 ± 13.6	0.006
Mean EPT(s) ± SD	12.2 ± 1.1	37.7 ± 5.7	0.0007
CCT Increase (µ)	67.4 ± 72.7	138.0 ± 103.1	0.018
Cell loss (%)	8.4 ± 8.1	15.8 ± 8.4	0.024
Postop VA > 6/ 18	19 (95%)	19 (90.5%)	1.00
*Phaco I = phacoemulsification in NS 2 + Cataract*;			
*Phaco = Phacoemulsification in white cataract*.			

**Fig. 2:**
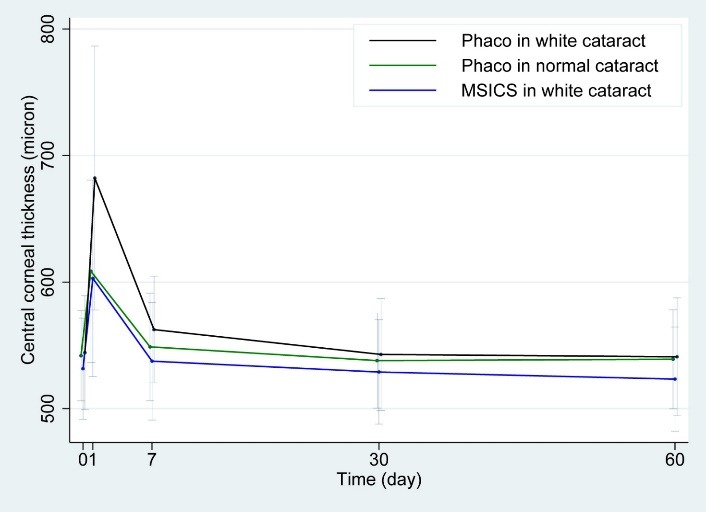
Central corneal thickness changes

Most MSICS and phaco patients had visual acuity worse than 3/ 200 to HM. The group who underwent MSICS averaged 13.2 minutes for the surgery. The first day after the surgery, the cornea, initially 531 microns thick, rose to 603 microns (an average increase of 73 microns); thickness returned to normal 1 month after the surgery. Endothelial cell loss 3 months post operation was at approximately 11.8% (**Table 3**, **Fig. 2**).

**Table 3:** Clinical outcomes

Clinical outcomes	MSICS (n = 21)		Phaco (n = 21)		
	X	SD	X	SD	p-value
CCT					
PRE	531.6	40.1	544.2	45.0	0.342
1 D	603.0	77.6	682.2	104.3	0.008
0.008	537.5	46.4	562.5	42.0	0.076
1 mo	528.9	41.2	542.9	44.3	0.299
CELL					
PRE					
1 mo	2540.2	218.4	2486.6	218.5	0.495
3 mo	2314.9	215.3	2214.1	347.6	0.265
	2239.4	232.5	2058.3	315.0	0.060
Cell loss (%)					
	11.8	6.4	15.8	8.4	0.111
*MSICS = Manual small-incision cataract surgery in white cataract*					
*Phaco II = Phacoemulsification in white cataract*					

For the group who underwent phaco, the operation time averaged 15.8 minutes. The cornea was initially 544 microns thick. The first day after surgery, it increased to 682 microns (an average increase of 138 microns); thickness returned to normal 1 month after the surgery. Endothelial cell loss 3 months post operation was about 15.8%.

The group who underwent phaco had significantly higher corneal thickness than the group who underwent MSICS (p< 0.008). Three months after the surgery, however, MSICS patients’ endothelial cell loss was 11.8%, while phaco patients’ endothelial cell loss was 15.8%, an insignificant difference (p< 0.111).

Regarding postoperative visual acuity, the patients who underwent MSICS had visibility higher than 6/ 18 at 71.4% and the patients who underwent phaco had visibility higher than 6/ 18 at 85.7% (p=0.454). Three months after the surgery, the MSICS patients had visibility higher than 6/ 18 at 90.5% and phaco patients had visibility higher than 6/ 18 at 90.5% (p=1.000), as shown in **Table 4**.

**Table 4:** Postoperative visual acuity

Postoperative visual acuity	MSICS (n = 21)		Phaco (n = 21)		
	n	%	n	%	p-value
Post VA 1 week					
< 6/ 60	1	4.8	0	0	0.454
6/ 60-6/ 18	5	23.8	3	14.3	
> 6/ 18	15	71.4	18	85.7	
Post VA 4 week					
< 6/ 60	1	4.8	0	0	1.000
1.000	1	4.8	2	9.5	
> 6/ 18	19	90.5	19	90.5	
*MSICS = Manual small-incision cataract surgery in white cataract*					
*Phaco = Phacoemulsification in white cataract*					

An intraoperative complication that occurred was a dropped nucleus in one patient from the group who underwent phaco; there were no occurrences of either posterior capsule rupture or dropped nucleus in the MSICS group.

## Discussion

White cataract can be treated by extracapsular cataract extraction, manual small incision cataract surgery, or phacoemulsification. The former surgery is currently not popular due to its large wound and long recovery time. As a result, MSICS and phaco have become more popular. Capsulorhexis is a challenging component of conducting white cataract surgery. However, with the development of a dyeing capsule [**16**-**19**], capsulorhexis has become easier. The difficulty level of the process for breaking the lens varies according to the hardness of the lens. If the lens is very hard, such as in a brown cataract, a lot of energy is required, and this can increase corneal thickness.

A comparative study of MSICS and phacoemulsification on 108 eyes found that corneal thickness increased by 9 micrometers and 70 micrometers in the MSICS group and the phaco group, respectively [**20**]. In the current study, the researcher performed cataract surgery on patients with normal lenses. This was useful as a benchmark to compare surgical skills. The operation time was 9.9 minutes and corneal thickness increased by 67 microns the first day after surgery, indicating that the standard performance was not different from that of a professional ophthalmologist [**15**]. After white cataract surgery, the corneal thickness increased by 67 microns and 138 microns in the MSICS group and the phaco group, respectively. This was a statistically significant difference (p=0.008).

A comparative study of MSICS and phacoemulsification on moderately hard cataracts found a 5.33% increase in corneal thickness (a 28 micron increase) and cell loss at 6.32% in the MSICS group and a 10.4% increase in corneal thickness (a 53 micron increase) and cell loss at 8.2% in the phaco group [**15**]. In addition, the white cataract patients lost endothelial cells at 11.8% and 15.8% post operation in the MSICS group and the phaco group, respectively. Cell losses were higher than those found in other studies due to the different degrees of white cataract hardness, ranging from moderate to high at NS 4+ levels. In cases of very hard cataracts, higher ultrasound energy is necessary, which can also result in higher corneal cell loss.

The limitations in this study were: 1) The lack of randomization in sampling could have led to bias in sample selections; however, according to the propensity score used for calculations, there were no differences between the sample groups; 2) The sample groups were small.

In summary, the current study showed that white cataract surgery using phacoemulsification caused higher levels of corneal thickness and endothelial cell loss than manual small incision cataract surgery.

**Acknowledgements**

The authors thank Jayanton Patumanond, MD, PhD, for assistance with statistical analyses and www.onlineproofreadingservices.net for editing the manuscript.

**Previous Presentation**: Data from the analysis were previously presented in part at the 31st Asia-Pacific Association of Cataract and Refractive Surgeons (APACRS) Congress, 19-21 July 2018 Chiang Mai, Thailand.

**Conflict of interest**

None.

**Thai clinical trial registry: TCTR20161129002.**
